# Changes in Health-Risk Behavior, Body Mass Index, Mental Well-Being, and Risk Status Following Participation in a Stepwise Web-Based and Face-to-Face Intervention for Prevention of Lifestyle-Related Diseases: Nonrandomized Follow-Up Cohort Study

**DOI:** 10.2196/16083

**Published:** 2020-07-09

**Authors:** Trine Thilsing, Anders Larrabee Sonderlund, Jens Sondergaard, Nanna Herning Svensson, Jeanette Reffstrup Christensen, Janus Laust Thomsen, Niels Christian Hvidt, Lars Bruun Larsen

**Affiliations:** 1 Research Unit of General Practice Department of Public Health University of Southern Denmark Odense Denmark; 2 Research Unit for General Practice in Aalborg Department of Clinical Medicine Aalborg University Hospital Aalborg Denmark

**Keywords:** health behavior, noncommunicable diseases, lifestyle-related disease, prevention, patient web portal, primary health care, risk reduction behavior

## Abstract

**Background:**

Recent evidence suggests the effectiveness of stepwise, targeted approaches for the prevention of lifestyle-related diseases with combinations of web-based and face-to-face interventions showing promising results.

**Objective:**

This paper reports on 1-year changes in health-risk behaviors, BMI, self-rated health, mental well-being, and risk of disease at 1-year follow-up after participation in a stepwise intervention that targeted persons at high risk of disease and persons with health-risk behavior. To this end, we distinguish between participants who took up the full intervention (web-based plus face-to-face) and those who received only the web-based intervention.

**Methods:**

The Early Detection and Prevention (Danish acronym: TOF) pilot study was conducted as a nonrandomized, 1-year follow-up intervention study in two municipalities in the Region of Southern Denmark. A total of 9400 citizens born between 1957 and 1986 (aged 29 to 60 years) were randomly sampled from participating general practitioner (GP) patient-list systems and were invited to take part in the study. Participants were subsequently stratified into risk groups based on their responses to a questionnaire on health-risk behavior and data from their GP’s electronic patient record (EPR) system. All participants received a digital personal health profile with individualized information on current health-risk behavior and targeted advice on relevant health-risk behavior changes. In addition, patients at high risk of disease, as indicated by their digital health profile, were offered a targeted intervention at their GP. Patients who were not deemed at high risk of disease but who exhibited health-risk behaviors were offered a targeted intervention at their municipal health center (MHC). At 1-year follow-up, health-risk behaviors, self-rated health, BMI, and mental well-being were reassessed by questionnaire, and current information on diagnoses and medical treatment was retrieved from the EPRs.

**Results:**

Of 598 patients at high risk of disease or with health-risk behavior, 135 took up the targeted intervention at their GP or MHC and 463 received the personal health profile only. From baseline to 1-year follow-up, the number of patients with unhealthy eating habits decreased, mean mental well-being increased, and smoking prevalence decreased in patients who had received the digital personal health profile alone. Among patients who took up the targeted intervention, unhealthy eating habits and sedentary lifestyles decreased and significant reductions in mean BMI were observed. At 1-year follow up, no health-risk behaviors were detected among 17.4% of patients who at baseline had exhibited health-risk behaviors or high risk of disease.

**Conclusions:**

A stepwise targeted preventive approach using web-based and face-to-face elements may lead to favorable lifestyle changes. Specifically, a web-based approach may improve smoking and eating habits and mental well-being, whereas supplementary face-to-face interventions may be necessary to improve exercise habits and BMI.

**Trial Registration:**

ClinicalTrials.gov NCT02797392; https://clinicaltrials.gov/ct2/show/NCT02797392

**International Registered Report Identifier (IRRID):**

RR2-10.1186/s12875-018-0820-8

## Introduction

Lifestyle-related diseases such as cardiovascular disease (CVD), type 2 diabetes mellitus, and chronic obstructive pulmonary disease (COPD) constitute a major health problem in most developed countries. A high overall prevalence of lifestyle-related diseases, combined with increases in the number of years lived with the resulting disease-related disabilities [1], represents a significant burden on any given health care system. As such, there is an urgent need to design and implement interventions that facilitate early identification and management of persons at risk of lifestyle-related diseases.

A systematic review from 2012 indicates that preventive health checks offered to the general population have no long-term effects on total mortality above and beyond those associated with standard care [2]. More recent systematic reviews of general practice–based health checks, however, suggest that people at high risk of chronic disease may benefit from targeted health checks [3,4]. In addition, a Cochrane review from 2011 showed that counseling- and education-based interventions targeting health risk behaviors can reduce mortality in the high-risk population [5]. Counseling and education may be delivered face to face, remotely (eg, by phone), or through web-based interventions [6-8], and evidence suggests that supplementing web-based interventions with face-to-face or remote counseling may increase the total effect of prevention programs [8].

In Denmark, the primary care sector is publicly funded and extensive, comprising municipal health centers (MHC) and general practitioner (GP) clinics. Almost all Danish citizens (98% of the population) are registered with a GP clinic [9]. The MHCs provide primary prevention (eg, smoking cessation and alcohol-reduction courses), while GPs are tasked with both primary and secondary prevention (eg, treatment for hypertension and hyperlipidemia) [9]. Targeted preventive actions are therefore an accepted and a well-integrated part of the Danish health care system. Nonetheless, these initiatives are often limited in terms of identifying the at-risk population.

In the Early Detection and Prevention project (TOF is the Danish acronym), we use a stepwise screening procedure to identify the at-risk population (ie, individuals at high risk of type 2 diabetes mellitus, COPD, or CVD and individuals who engage in health-risk behaviors). All screened individuals receive a digital personal health profile containing individualized information on current health-risk behaviors, risk of disease, and relevant preventive health services. In addition, individuals at high risk of the aforementioned diseases and those with health-risk behaviors are offered a targeted intervention at their GP or MHC. The GP intervention comprises a focused clinical examination and health dialog. At the MHC, participants are invited to one or two health dialogs. The overall purpose of the TOF intervention is to encourage and support participants to change their health-risk behavior, initiate preventive treatment if needed, and promote health and longevity. The TOF intervention is described in detail in a study protocol article [10].

In line with the Medical Research Council guidelines on complex interventions, the interventions were pilot-tested for acceptability, feasibility, and short-term effects in two municipalities in 2016 [10,11].

This paper reports on changes in health-risk behaviors, BMI, self-rated health, mental well-being, and risk of disease from baseline to 1-year follow-up among persons at high risk of disease and persons with health-risk behaviors. To this end, we distinguish between persons who take up the targeted interventions at their GP or MHC and those who receive the digital personal health profile but forego the targeted interventions.

## Methods

### Setting and Design

The TOF pilot study was carried out as a nonrandomized, 1-year follow-up cohort study in two Danish municipalities (Varde and Haderslev; total population, January 2016: 106,318).

### Population

The study population comprised patients randomly sampled from participating GPs’ patient list system. Almost all Danish citizens are registered with a GP [9,12,13], and each GP has an average of approximately 1600 registered patients. This study included patients born between 1957 and 1986 (age 29 to 60 years). A total of 200 eligible patients were randomly selected per GP. Patients who resided outside of the participating municipalities and patients who did not have a digital mailbox were excluded from the study. A digital mailbox is provided by the Danish government for secure and direct communication between citizens and public authorities. In general, all permanent citizens are obliged to have a digital mailbox, but citizens with low information technology literacy (usually elderly persons), cognitive impairment, or other complicating factors may opt out of the digital mail system.

### Recruitment and Baseline Questionnaire

In January 2016, GPs residing in the two municipalities were invited to take part in the study. In April 2016, the study population was sampled and an invitation and informed consent form were sent to prospective participants by digital mail. The consent form covered participation and the retrieval of relevant diagnoses and medical scripts from the GPs’ electronic patient record (EPR) systems. This information was used to identify patients who were registered with International Classification of Primary Care–2 codes or medical scripts related to CVD, type 2 diabetes mellitus, COPD, hypertension, or hyperlipidemia. In September 2016, all participants received a digital questionnaire with items on height, weight, self-rated health, family history of diabetes, known hypertension, COPD-related symptoms, smoking habits, leisure activity level, alcohol intake, and eating habits. The questionnaire items were from the Danish Diabetes Risk model [14], the COPD population screener [15], the HeartScore BMI score for CVD [16], the Swedish National Guidelines on Disease Prevention [17], and the Danish National Health Profile [18]. In addition, mental well-being was assessed using the Short Warwick-Edinburgh Mental Well-Being Scale (SWEMWBS) [19]. Both the initial invitation and questionnaire were sent on behalf of the patients’ GP and the MHC.

### Stratification of Patients

Based on information from the GPs’ EPR systems and the questionnaire results, patients were stratified to one of four groups (see Table 1). Group 1 included patients with preexisting diagnoses and/or treatment for type 2 diabetes mellitus, CVD, hypertension, hyperlipidemia, and/or COPD. These patients were identified solely from the EPR information. Group 2 comprised patients who were deemed at high risk for type 2 diabetes mellitus, CVD, or COPD based on three validated risk scores [14-16]. Next, patients who were not at high risk of disease but who engaged in health-risk behaviors were placed in group 3. Health-risk behaviors included current smoking, consuming more than 14/21 (female/male) standard units of alcohol per week, having an unhealthy diet (diet score ≤4 on a 12-point scale drawn from the Swedish National Guidelines on Disease Prevention) [17], maintaining a BMI ≥35 kg/m^2^, and/or engaging in a generally sedentary lifestyle as defined by the Saltin-Grimby Physical Activity Level Scale (primary leisure time activity level during the past year: reading, watching television, or other sedentary activities) [20]. Finally, group 4 comprised patients with no health-risk behaviors and no need for further intervention.

Patients at high risk of disease (group 2) and patients with health-risk behaviors (group 3) were eligible to receive the full TOF intervention. These two groups are therefore the focus of this paper.

### TOF Intervention

The TOF intervention included a digital personal health profile and targeted preventive activities at patients’ GP or MHC. The digital personal health profile was designed to encourage patients to change their health-risk behavior and follow the tailored advice provided by the system. The health profile included individualized information on current health-risk behaviors and risk of disease, personalized advice on relevant health-risk behavior changes, and information about relevant preventive health services. Participants could access their personal health profile on a password-protected website [10].

The preventive activities at the GPs or MHCs targeted patients with different risk profiles. Patients at high risk of disease (group 2) were offered a clinical examination at their GP, including measurements of glycated hemoglobin, cholesterol, height, weight, and blood pressure, plus lung functions and electrocardiogram, if indicated, and a subsequent health dialog, scheduled in 30-minute time slots. Patients with health-risk behaviors (group 3) were advised to consult their MHC for a 15-minute telephone-based health dialog. The health dialog could be requested by patients on their personal health profile. If necessary, the telephone-based health dialog was followed up with a 1-hour face-to-face consultation at the MHC.

All patients offered a health dialog, either at their GP or at their MHC, were encouraged to prepare by answering questions about known determinants of behavior change (eg, motivation, resources, social network, mental health, former experience with behavior change) [21,22]. This information, along with information about health-risk behavior, was shared with health professionals on separate user interfaces of the digital support system. During the health dialog, the patient and health professional would work together to develop a prevention plan that set a goal for health-risk behavior change and determined the necessary means for achieving that goal. The prevention plan was subsequently registered on the digital support system by the health professional and was thus accessible to both health professional and patient. If relevant and feasible, the patient would be referred to municipal behavior change interventions (such as smoking cessation courses, exercise classes, etc), prescribed medical treatment by their GP, or both.

The design of the digital support system was inspired by the work of Krist and colleagues’ research on preventive EPRs and by the results of a Delphi process completed to identify factors for optimal development of health-related websites [23-25]. Details about the digital personal health profile and the digital support system are published elsewhere [10].

The TOF intervention was available from September through December 2016. However, intervention-initiated referrals to municipal health-risk behavior change interventions and prescription of medical treatment continued beyond this time frame.

**Table 1:** Group characteristics and preventive activities offered to participants in the high-risk of disease group and the health-risk behavior group (groups 2 and 3).

Variables	High risk of disease group	Health-risk behavior group
Group characteristics	Patients at high risk of type 2 diabetes mellitus, CVD^a^, or COPD^b^	Patients with health-risk behaviors such as current smoking, high-risk alcohol intake, sedentary lifestyle, unhealthy diet and/or maintaining a BMI ≥35 kg/m^2^
Intervention offered	Digital personal health profile, focused clinical examination, and subsequent 30-minute health dialog at the GP^c^	Digital personal health profile, 15-minute telephone-based health dialog, and optional 1-hour face-to-face consultation at the MHC^d^

^a^CVD: cardiovascular disease.

^b^COPD: chronic obstructive pulmonary disease.

^c^GP: general practitioner.

^d^MHC: municipal health center.

### Follow-Up Questionnaire and Electronic Patient Record Information

In September 2017, 1 year after baseline assessments, all consenting patients received a follow-up electronic questionnaire that included the same items on weight, health-risk behaviors, self-rated health, COPD-related symptoms, and mental well-being as the baseline questionnaire. In addition, up-to-date EPR information was collected to identify any patients who had been diagnosed with or commenced medical treatment for type 2 diabetes mellitus, CVD, hypertension, hyperlipidemia, or COPD during the 1-year follow-up period.

### Outcomes and Statistical Analysis

We report on specific health-risk behavior changes observed between baseline and 1-year follow-up in patients at high risk of disease and patients with health-risk behaviors.

Health-risk behaviors were treated as dichotomous variables (yes/no): current smoking (including daily and occasional smoking), high-risk alcohol intake (ie, above 14/21 [female/male] standard units of alcohol per week), unhealthy diet (ie, diet score ≤4 on a 12-point scale drawn from the Swedish National Guidelines on Disease Prevention) [17], and sedentary lifestyle (according to the Saltin-Grimby Physical Activity Level Scale [20]). We also looked at changes in self-rated health (“In general, how would you rate your health at present?” with response categories excellent, very good, good, fair, and poor, dichotomized into two categories: fair or poor and good, very good, or excellent), BMI, and mental well-being (from SWEMWBS) from baseline to 1-year follow-up [26]. The raw SWEMWBS score was converted to a metric score using a conversion table.

We report on these changes among participants who took up the targeted interventions at their GP or MHC and participants who received only the digital personal health profile. Attending the targeted intervention at the GP was defined as having received the focused clinical examination, whereas attendance at the MHC was defined as having participated at minimum in the telephone-based health dialog.

Changes in health-risk behaviors and self-rated health from baseline to 1-year follow-up were analyzed using a McNemar test. Changes in BMI and SWEMWBS scores were analyzed using paired *t* tests. Analyses were repeated after stratifying by gender.

Finally, we analyzed any changes in individual risk stratification from baseline to 1-year follow-up. Stratification groups at 1-year follow-up were determined from up-to-date data on health-risk behaviors, BMI, diagnoses, and medical scripts from the EPR system. The follow-up calculations were performed as described in the stratification of patients section except we applied baseline age to the three validated risk scores in order to preclude age-related changes in risk groups. That is, we investigated whether patients were reallocated during the study to another risk group than the one at baseline by virtue of changes in diagnoses, health-risk behaviors, or BMI rather than age.

Nonresponse bias was assessed by comparing baseline characteristics of participants answering the 1-year follow-up questionnaire with those who did not. Unadjusted estimates were generated from Fisher exact tests for dichotomous variables and *t* tests for continuous variables. These estimates were adjusted for age and gender differences using logistic and linear regression, respectively. The distribution of SWEMWBS scores and BMI were assessed for normality by visual inspection of histograms. There were no missing values for health-risk behaviors, BMI, or self-rated health as participants responses to these questions were compulsory. Statistical significance was set at *P*<.05. Statistical analyses were performed using Stata 15.1 (StataCorp LLC) statistical software for Windows.

### Ethics Approval and Consent to Participate

The study was approved by the Danish Data Protection Agency (J.nr. 2015-57-00089) and registered with the University of Southern Denmark’s list of approved studies (J.nr. 10.361) and on ClinicalTrials.gov [NCT02797392]. According to Danish regulations, the study did not need approval from a health research ethics committee. The study complies with the World Medical Association Declaration of Helsinki, including providing informed consent to study participation and disclosure of data from the GP EPRs.

## Results

**Figure 1:**
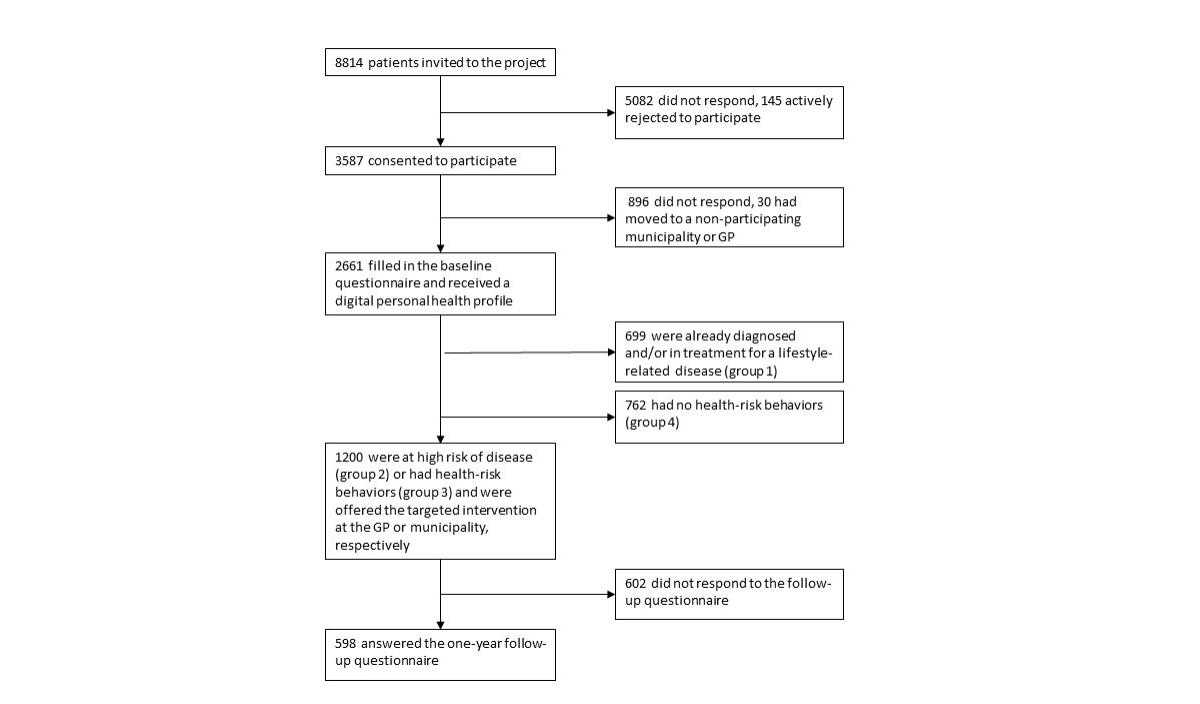
Flowchart of the TOF pilot study.

A total of 69% (47/68) of invited GPs agreed to participate in the study, resulting in a source population of 9400 patients. Among these, 586 were excluded because they resided outside the participating municipalities or did not have a digital mailbox. Of the 8814 patients who received the initial invitation, 3587 patients consented to participate, and 2661 subsequently completed the baseline questionnaire and received a digital personal health profile [27]. Of these, 582 were deemed to be at high risk (group 2) and were offered the targeted intervention at their GP. Another 618 patients engaged in health-risk behaviors (group 3) and were offered the targeted intervention at the MHC [28]. At 1-year follow-up, 56.2% (327/582) of patients from the high-risk group and 43.9% (271/618) from the health-risk behavior group responded to the questionnaire (Figure 1). Of these, 135 (77 women, 58 men) had attended the targeted interventions at their GP or MHC.

Table 2 shows the baseline characteristics of the participants who responded to the follow-up questionnaire and those who did not. The follow-up group was older than the group that did not answer the follow-up questionnaire (48.8 vs 46.5 years, *P*<.001), included more men (52.5% vs 45.7%, *P*=.02), and had fewer current smokers (21.6% vs 31.7%, *P*<.001). The two groups did not differ on any of the other items.

For participants who received the digital personal health profile only, a significant reduction in the number of current smokers and participants with unhealthy eating habits was seen from baseline to 1-year follow-up (Table 3). In addition, the mental well-being score was significantly higher at 1-year follow-up compared with baseline levels. Specifically, 40.0% (183/457) of participants experienced an increase of one or more in their mental well-being score. In subgroup analyses, changes in mental well-being reached statistical significance in women, whereas decreases in current smoking prevalence was statistically significant for men only.

No significant changes in the prevalence of sedentary behavior, high-risk alcohol intake, or fair/poor self-rated health were observed among participants who received only the digital personal health profile. The number of participants with a BMI >30 kg/m^2^ decreased from 25.5% (118/463) at baseline to 24.0% (111/463) at 1-year follow-up, but no significant changes in mean BMI were detected.

Among participants who attended the targeted intervention at their GP or MHC, a similar drop in the number of participants with unhealthy eating habits was observed (Table 4). In addition, mean BMI and the number of participants with a sedentary lifestyle had declined at 1-year follow-up, although subgroup analyses were statistically nonsignificant. The number of participants with a BMI >30 kg/m^2^ decreased from 34.1% (46/135) at baseline to 27.4% (37/135) at 1-year follow-up. No significant changes were observed in mental well-being, current smoking status, high-risk alcohol intake, or self-rated health.

**Figure 2:**
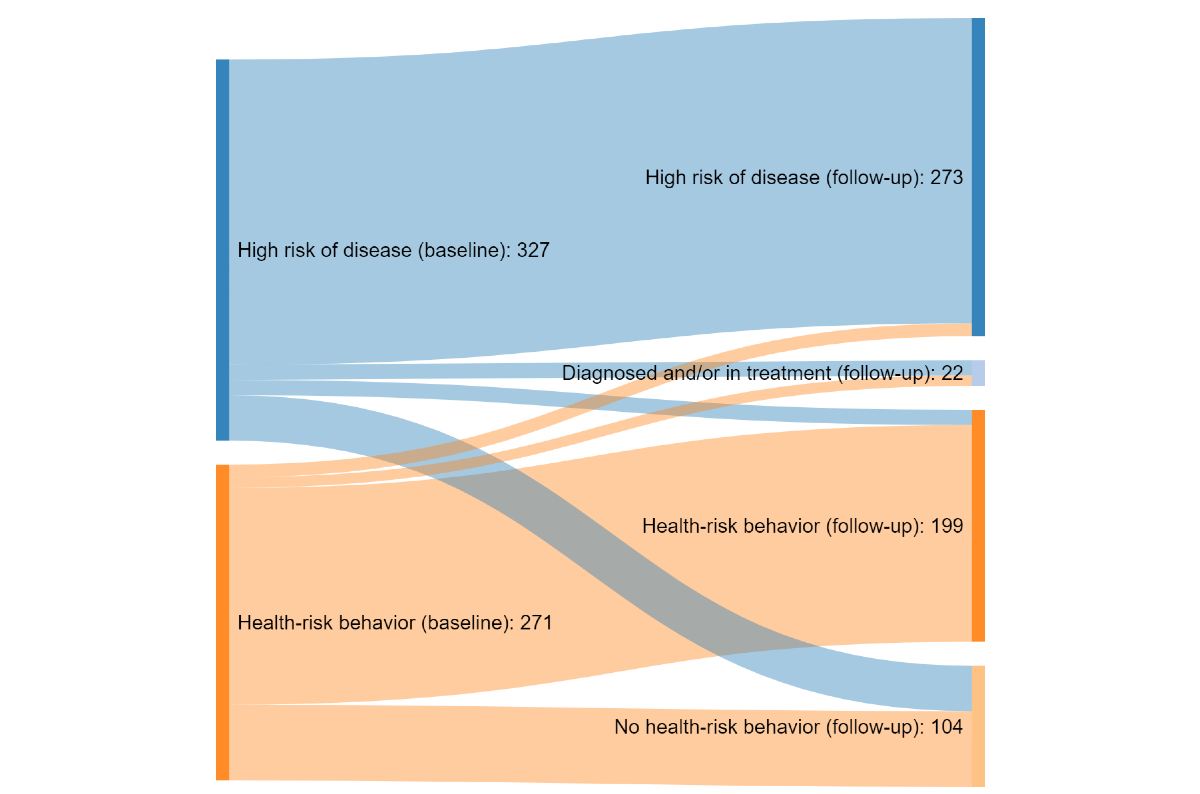
Participant change in risk status from baseline to 1-year follow-up.

Figure 2 shows the changes in risk status from baseline to 1-year follow-up. Among 327 participants at high risk of disease and 271 participants with health-risk behaviors, 39 (11.9%) and 65 (24.0%), respectively, had no health-risk behaviors at 1-year follow-up. A total 4.0% (13/327) of participants at high risk of disease and 3.3% (9/271) with health-risk behaviors were diagnosed with or commenced preventive medical treatment for hypertension, hyperlipidemia, CVD, type 2 diabetes mellitus, or COPD between baseline and 1-year follow-up. In addition, 4.1% (11/271) of participants with health-risk behaviors were at high risk of disease at 1-year follow-up, whereas 4.0% (13/327) had reduced their risk status from high risk of disease to health-risk behaviors.

**Table 2:** Baseline characteristics among high-risk and health-risk behavior participants with and without 1-year follow-up (n=1200).

Variable	High-risk and health-risk behavior participants (groups 2 and 3)				
	With follow-up (n=598)	Without follow-up (n=602)	*P* unadjusted	*P* adjusted^a^	All (n=1200)
Age in years, mean (SD)	48.8 (8.2)	46.5 (8.4)	<.001	N/A^b^	47.7 (8.4)
Gender, male, n (%)	314 (52.5)	275 (45.7)	.02	N/A	589 (49.1)
Current smoker, n (%)	129 (21.6)	191 (31.7)	<.001	.001	320 (26.7)
Unhealthy diet, n (%)	217 (36.3)	252 (41.9)	.05	.41	469 (39.1)
Sedentary lifestyle, n (%)	125 (20.9)	134 (22.3)	.58	.69	259 (21.6)
High-risk alcohol intake, n (%)	19 (3.2)	28 (4.7)	.23	.09	47 (3.9)
BMI (kg/m^2^), mean (SD)	27.7 (5.5)	27.6 (5.5)	.63	.50	27.6 (5.5)
Fair or poor self-rated health, n (%)	80 (13.4)	78 (13.0)	.87	.53	158 (13.2)
Mental well-being score^c^, mean (SD)	24.2 (3.6)	24.0 (3.7)	.43	.96	24.1 (3.6)

^a^Adjusted for age and gender differences.

^b^Not applicable.

^c^A total of 1176 (591 with follow-up and 585 without) answered the questions on mental well-being.

**Table 3:** Health-risk behaviors, BMI, self-rated health, and mental well-being at baseline and 1-year follow-up among high-risk and health-risk behavior participants receiving the digital personal health profile only.

Variable	All (n=463)			Women (n=207)			Men (n=256)		
	Baseline	1-year follow-up	*P* value	Baseline	1-year follow-up	*P* value	Baseline	1-year follow-up	*P* value
Current smoker, n (%)	106 (22.9)	93 (20.1)	.003	54 (26.1)	49 (23.7)	.10	52 (20.3)	44 (17.2)	.01
Unhealthy diet, n (%)	161 (34.8)	128 (27.7)	<.001	58 (28.0)	44 (21.3)	.01	103 (40.2)	84 (32.8)	.03
Sedentary lifestyle, n (%)	92 (19.9)	77 (16.6)	.07	51 (24.6)	40 (19.3)	.06	41 (16.0)	37 (14.5)	.48
High-risk alcohol intake, n (%)	15 (3.2)	13 (2.8)	.56	<5	<5	.99	11 (4.3)	9 (3.5)	.48
BMI (kg/m^2^), mean (SD)	27.4 (5.5)	27.3 (4.8)	.36	27.6 (6.1)	27.7 (5.8)	.88	27.3 (5.0)	27.0 (3.8)	.19
Fair or poor self-rated health, n (%)	57 (12.3)	62 (13.4)	.51	31 (15.0)	34 (16.4)	.58	26 (10.2)	28 (10.9)	.71
Mental well-being score^a^, mean (SD)	24.3 (3.6)	24.7 (4.2)	.01	24.3 (3.9)	24.9 (4.4)	.04	24.3 (3.3)	24.6 (4.0)	.11

^a^SWEMWBS score: a total of 457 (203 women and 254 men) answered the questions on mental well-being.

**Table 4:** Health-risk behaviors, BMI, self-rated health, and mental well-being at baseline and 1-year follow-up among high-risk and health-risk behavior participants who received the digital personal health profile and targeted intervention at their general practitioner or municipal health center.

Variable	All (n=135)				Women (n=77)				Men (n=58)		
	Baseline	1-year follow-up	*P* value	Baseline		1-year follow-up	*P* value	Baseline		1-year follow-up	*P* value
Current smoker, n (%)	23 (17.0)	21 (15.6)	.48	12 (15.6)		13 (16.9)	.56	11 (19.0)		8 (13.8)	.18
Unhealthy diet, n (%)	56 (41.5)	39 (29.9)	.001	28 (36.4)		19 (24.7)	.007	28 (48.3)		20 (34.5)	.046
Sedentary lifestyle, n (%)	33 (24.4)	17 (12.6)	.001	21 (27.3)		13 (16.9)	.03	12 (20.7)		n<5	.01
High-risk alcohol intake, n (%)	<5	<5	.65	<5		<5	.32	<5		<5	.32
BMI (kg/m^2^), mean (SD)	28.7 (5.5)	28.3 (5.7)	.02	29.6 (5.8)		29.1 (6.1)	.11	27.6 (4.9)		27.2 (4.9)	.05
Fair or poor self-rated health, n (%)	23 (17.0)	21 (15.6)	.64	18 (23.4)		14 (18.2)	.25	5 (8.6)		7 (12.1)	.41
Mental well-being^a^, mean (SD)	24.0 (3.6)	24.4 (4.2)	.17	24.0 (3.6)		24.2 (4.4)	.50	24.0 (3.8)		24.7 (3.9)	.20

^a^SWEMWBS score: a total of 134 (77 women and 57 men) answered the questions on mental well-being.

## Discussion

### Principal Findings

Our results suggest that a stepwise and targeted prevention approach focusing on patients at high risk of lifestyle-related diseases and patients with health-risk behaviors may be effective in promoting certain healthy lifestyle changes.

Significant improvements in smoking and eating habits and mental well-being were seen among patients who received the web-based intervention. Supplementary face-to-face intervention, however, appeared to be necessary to significantly improve exercise habits and BMI.

Improvements in dietary habits were observed among participants who attended the targeted interventions at their GPs and MHCs and among participants who got a digital personal health profile only. These findings are in line with previous evidence on the effectiveness of primary-care-based lifestyle interventions [29-32] and exclusively web-based interventions [6].

Similarly, the observed reduction in current smoking status among participants who got a digital personal health profile is consistent with previous evidence from web-based smoking cessation interventions [7]. Although brief smoking interventions delivered in general practice and in the primary care sector have previously been shown to be successful [33,34], smoking prevalence was unchanged at 1-year follow-up in patients who participated in the targeted intervention. This result may in part be attributed to the limited sample size and the low follow-up response rate among baseline current smokers. In addition, more nonsmokers than current smokers took up the targeted intervention at their MHC [28].

The observed improvements in mental well-being and concomitant healthy lifestyle changes are consistent with findings from other lifestyle intervention studies [35,36]. Specifically, a systematic review concluded that smoking cessation was associated with reduced depression, anxiety, and stress as well as improved mood and quality of life [37]. Such results may in part be explained by biological mechanisms, as smoking causes alterations in the nicotine pathways in the brain, which has been associated with depressed mood and anxiety [38]. In addition, epidemiological studies have revealed close associations between fruit and vegetable consumption and mental health [39], with some studies even suggesting a causal relationship [40,41]. Although the changes in mean SWEMWBS score were relatively small in this study, 40.0% of the participants experienced improvements exceeding the suggested threshold for statistically meaningful change at the individual level [42]. Despite improvements in mean SWEMWBS score among participants attending the targeted interventions, the changes did not reach statistical significance. Although possibly attributable to the small sample size, these findings are somewhat surprising as additional human support during targeted interventions should intuitively facilitate mental health. However, participants attending the targeted intervention at their GP had lower baseline self-efficacy than those who received the digital personal health profile alone [28]. This may have influenced the results as self-efficacy is known to be associated with well-being [43].

Significant reductions in BMI were seen among participants attending the targeted intervention but not among those who received only the digital personal health profile. This may be due to higher motivation among those participants who chose to take up the targeted interventions at their GP or MHC. In comparison, a recent systematic review indicated that significant weight reductions could be achieved through web-based interventions alone. However, the review also showed that blended interventions (ie, combination of an internet application and human support) like the one tested in this study were more effective in reducing weight than purely web-based ones [6]. The reduction in BMI fits well with the concurrent improvements in physical activity and dietary habits. Although improved, BMI changes did not reach statistical significance in subgroup analyses. This may be attributable to the small sample size.

The behavior change techniques (BCTs) applied in the TOF study are partially inspired by tried and tested methods from dietary interventions. These include goal setting (outcome), plan social support/social change, social comparison, and barrier identification/problem solving [30,44]. These BCTs might have contributed to the positive effect on dietary habits. In addition, information on the consequences of behavior in general, which has been associated with a positive change in physical activity level, was incorporated into the digital personal health profile [45]. A recent review identified interventions encouraging self-monitoring of behavioral outcomes or using follow-up prompts to be the most effective in maintaining physical activity improvements [46]. Such BCTs were not used in the TOF pilot study but may well be relevant in future effectiveness studies.

Results from a recent systematic review on medium-intensity (31 to 360 minutes) to high-intensity (>360 minutes) behavioral counseling in high-risk populations showed improvements in dietary intake and physical activity as well as concordant reductions in intermediate CVD outcomes such as total cholesterol, low-density lipoprotein cholesterol, blood pressure, fasting glucose, diabetes incidence, and weight outcomes [47]. In our study, 17.4% (104/598) improved their lifestyle to the extent that no health-risk behaviors were present 1 year after entering the study, and 19.6% (117/598) had changed to a lower risk group at 1-year follow-up. We believe such changes are likely to improve intermediate CVD outcomes like the ones described above (not included in this study). Effects in terms of intermediate outcomes such as changes in the level of biomarkers and incidence of disease should be examined further.

### Strengths and Limitations

This study used validated questions and risk scores to assess health-risk behaviors and risk of disease and used a longer follow-up period than most lifestyle intervention studies. Health-risk behavior changes were assessed by self-reported outcomes, which may be subject to reporting bias. However, participants were not asked if they had improved their lifestyle but merely responded to the same questions on health-risk behaviors at baseline and at 1-year follow-up. We therefore believe the risk of social desirability bias to be minimal. In this study, the follow-up response rate was 50%, which may affect the generalizability of the results. In addition, responders differed from nonresponders by being older and more often male. Such differences may point to more unfavorable health-risk profiles among responders [48], but the two groups only differed in current smoking status. Finally, the study did not include an untreated control group. Therefore, the observed changes in health-risk behaviors could be partly attributable to factors other than the intervention tested.

### Conclusion

Results from this pilot study indicate that persons at high risk of disease and persons with health-risk behaviors may benefit from a stepwise, targeted intervention in terms of favorable lifestyle changes. Specifically, a web-based approach may improve smoking and eating habits and mental well-being, whereas supplementary face-to-face interventions may be necessary to improve physical exercise habits and BMI. While the extent of effects reported here seem to depend on the breadth of intervention received, it is important to note that even a low-cost, web-based intervention alone may be effective in facilitating meaningful health behavior change. Long-term effects need to be assessed in a large-scale, controlled study design.

## Abbreviations

BCT: behavior change technique
COPD: chronic obstructive pulmonary disease
CVD: cardiovascular disease
EPR: electronic patient record
GP: general practitioner
MHC: municipal health center
SWEMWBS: Short Warwick-Edinburgh Mental Well-Being Scale
TOF: Early Detection and Protection Project (Danish)
